# Bovine Viral Diarrhea Virus in Zoos: A Perspective from the Veterinary Team

**DOI:** 10.3389/fmicb.2015.01496

**Published:** 2016-01-06

**Authors:** Jack J. Kottwitz, Melissa Ortiz

**Affiliations:** ^1^Department of Anatomy, Physiology and Pharmacology, College of Veterinary Medicine, Auburn UniversityAuburn, AL, USA; ^2^Wildlife Conservation Society—Queens ZooFlushing, NY, USA

**Keywords:** bovine viral diarrhea virus, exotic ruminant, zoo, persistent infection, abortion

## Abstract

The many different species in close proximity make zoological collections a unique environment for disease transmission. Bovine Viral Diarrhea Virus (BVDV) is of special concern with zoos due to the numerous exotic ruminant species that this virus can infect. BVDV occurs as both a non-cytopathic and a cytopathic strain both of which are capable of infecting exotic ruminants. The cytopathic strain causes mucosal disease (MD) and death. Infection with the non-cytopathic strain may produce persistently infected (PI) animals. PI individuals may show vague clinical signs, including abortion. Management of BVDV in zoos should focus on identification of PI individuals and prevention of infection of other animals of the collection. Variability makes serological testing as the sole method of screening for BVDV infection undesirable in exotic ruminants. Combination testing provides a definitive answer, especially in sensitive wildlife. Use of a combination of antigen-capture ELISA (ACE) with haired skin, Real Time-PCR (RT-PCR) on whole blood, and antibody detection via serum neutralization has the greatest potential to identify PI animals. An animal that is positive on both ACE and RT-PCR, but is negative on serology should be considered highly suspicious of being a PI, and should be isolated and undergo repeat testing 4–6 weeks later to confirm positive status. This testing methodology also allows screening of pregnant and newborn animals. Isolation or culling may need to be considered in animals determined to be positive via combination testing. These decisions should only be made after careful consideration and evaluation, especially with endangered species.

## Introduction

Pestiviruses, within the family Flaviviridae, are viruses that may persist undiagnosed in exotic animals and zoological collections. To understand how this happens, and the unique concerns when addressing zoo animals, one must first understand some of the nuances of zoos and zoo collection management. There are few places in the natural world that rival the species density and diversity as modern zoos and wild animal parks. Interactions may occur in species that have overlapping natural environments, but rarely encounter each other in the wild. Modern zoos also serve as breeding facilities for endangered wildlife, and as a result may unintentionally provide perpetuating sources of infection or reservoir hosts for pathogens due to attempts to breed from very limited founder populations (Mohamed, 2015).

Most public zoos in America and Europe have experienced a positive shift over the last 30 years away from traditional bare, concrete floored, and chain-link fence enclosures. These traditional displays allowed easy viewing by zoo patrons but may have further perpetuated the spread of diseases by increasing animal stress, which may result in weakened immune function, along with exposure to feces and other contaminants that can serve as sources of infection. Unfortunately, there are still privately owned facilities where animals exist in these conditions. The mixing of species and space limitations in a zoo can create a unique environment for transmission of diseases. Traditional mixed species displays, such as an “African Savannah” or “Asian Rainforest,” combining different species can inadvertently serve as sources of infection to novel hosts. Newer management practices, such as rotational displays designed to allow movement between separate, but shared display areas through the day have maximized space and environmental enrichment for zoo animals, but may also result in direct and indirect mixing of species in a manner that results in exposure to novel diseases (Nolen, 2002; Coe, 2003).

Zoo veterinarians must remain vigilant for disease causing organisms capable of infecting multiple hosts. Obviously, the viral and bacterial diseases usually receiving the most attention for routine surveillance and prevention are those capable of causing zoonotic infection in humans. Infection of any zoo animal with a reportable disease having public health importance strikes fear in all associated zoo veterinarians and zoo administration (National Notifiable Diseases Surveillance System, 2015; Backues et al., 2011). Traditional estimates of disease occurrence have attributed as much as 75% of human emerging infectious diseases to zoonotic pathogens obtained from exposure to animals and wildlife (Taylor et al., 2001). Zoonotic diseases in zoos are local headline news stories even if there is no risk of zoo patron exposure and become national news if there is a human infection (Science on NBC News.com, 2005; Ganucheau, 2015). The American Association of Zoo Veterinarians (AAZV) and Association of Zoos and Aquariums and similar organizations in Canada, Europe, and Australia maintain quarantine guidelines for the introduction of new animals, but those quarantine guidelines are designed to be flexible, allowing for nuances of individual collections (Backues et al., 2011; Association of Zoos and Aquariums, 2015). These organizations also maintain specific guidelines for human interaction with zoo animals, to prevent human exposure (Backues et al., 2011; Association of Zoos and Aquariums, 2015).

The intervention of public health authorities, including veterinarians, for management of a disease outbreak reportable to government agencies may alleviate some of the pressure on zoo management for animal care decisions. However, this may further complicate the management of a disease outbreak in a zoo because normal protocols for isolation and eradication of infection in domestic animals, such as dramatic depopulation for an avian influenza outbreak in a broiler chicken farm, may be unreasonable for a zoo displaying rare, or endangered animals. This delicate balance of surveillance and identification of reportable diseases, combined with the ever looming presence of public perception of appropriate zoo management, means that diseases may go undetected for long periods of time, slowly brewing in the zoo collection for years, before being properly identified. Despite these concerns, general goals of all zoo health programs should involve steps that insure: (1) protecting the visiting public from disease exposure while protecting the animals from exposure from humans (2) protecting the collection from disease exposure from each other and (3) preventing the introduction of disease into regions around the zoo.

## Pestiviruses

Pestiviruses have been historically classified into four phylogenetic groups: bovine viral diarrhea virus serovar 1 (BVDV-1), bovine viral diarrhea virus serovar 2 (BVDV-2), classical swine fever virus (CSFV), and border disease virus (BDV) of sheep (Vilcek et al., 2000). The identification of novel strains of BVDV, including giraffe-1 and reindeer-1 have occurred within the last 15 years, however much remains unknown about these viruses (Avalos-Ramirez et al., 2001). While classical swine fever virus has been eradicated from many developed countries, CSFV remains a disease immediately reportable in many countries due to its potential economic impact (Classical Swine Fever Surviellence Plan USDA, 2007). Border disease has been recognized in most sheep-rearing areas of the world. However, it along with BVDV, remain diseases that may not be immediately reportable to government agencies or other authorities (Vilcek et al., 2000; Classical Swine Fever Surviellence Plan USDA, 2007).

BVDV is of concern to zoos because of the variety of species in the Order Artiodactyla, and suborder Ruminatia, including *Cervidae, Antilocapridae*, and *Bovidae*, with confirmed infections. Specifically, BVDV has been documented in wild and captive populations of mule deer (*Odocoileus hemionus*), white-tailed deer (*Odocoileus virginianus*), elk (*Cervus elaphus*), Japanese serow (*Capricornis crispus*), Canadian bison (*Bison bison bison*), water buffalo (*Bubalis bubalis*), roe deer (*Capreolus capreolus*), mouse deer (*Tragulus javanicus*), red deer (*Cervus elaphus*), bongo (*Tragelaphus eurycerus*), eland (*Taurotragus oryx*), wildebeest (*Connochaetes sp*.), nilgai (*Boselaphus tragocamelus*), axis deer (*Axis axis*), and barasingha deer (*Cervus duvaucelii*) (Doyle and Heuschele, 1983; Becher et al., 1999; Tessaro et al., 1999; Deregt et al., 2005; Craig et al., 2008). BVDV has also been isolated from animals frequently found in petting zoos including domestic cattle, alpaca, sheep, and goats (Pratelli et al., 2001; Scherer et al., 2001; Mattson et al., 2006; Mishra et al., 2007). All of these species are commonly found in modern zoos, may act as a reservoir for infection of other captive ruminants by BVDV.

## BVDV in zoo animals

Because BVDV infections in domestic cattle herds can cause significant economic losses, this virus has been the subject of intense research in domestic cattle. The pathogenesis of BVDV appears to be similar in domestic and wild ruminants, however specifics of individual species infection requires additional research. In domestic cattle, two viral genotypes (BVDV-1 and BVDV-2) have been identified, and cytopathic and non-cytopathic biotypes are described according to their effects in cell culture (Gamlen et al., 2005; Vilcek et al., 2005). The non-cytopathic biotype of BVDV is capable of establishing persistent infections (PI) in a fetus when the dam is infected during a specific window of gestation. This occurs when an immunologically naıve cow is infected with a non-cytopathic viral strain between 45 and 125 days of gestation (Brock, 2003; Gamlen et al., 2005; Peterhans et al., 2010). It is presumed that infected wild ruminants will have a similar infective window during the first one-third of gestation. However, those points in time have not yet been clearly defined experimentally in any species other than white tailed deer, where infection occurring between days 45 and 52 of gestation result in a PI fawn (Passler et al., 2007). This specific window of time predates immune system maturation, allowing the virus to remain in the affected fetus. Such fetuses may develop normally, and can be born apparently healthy, but will remain infected with the virus for life (Brock, 2003; Passler et al., 2007; Peterhans et al., 2010).

In addition to domestic cattle, persistent BVDV infection has been identified in domestic sheep and alpaca, which are common inhabitants of petting zoos (Carman et al., 2005; Mattson et al., 2006). There are also reports of definitive natural and experimental persistent infection in zoo animals and wildlife, including mouse deer, white tailed deer, North American elk, mountain goats, and eland (Tessaro et al., 1999; Vilcek et al., 2000; Uttenthal et al., 2005; Passler et al., 2007, 2009, 2010; Nelson et al., 2008). Animals infected with non-cytopathic BVD strains are considered immunotolerant to the infecting viral strain, and as a result are unable to clear the virus (Brock, 2003; Passler et al., 2007). PI neonates will heavily shed virus throughout their lives, creating a significant reservoir for infection, especially in a closed herd (Brock, 2003; Shoemaker et al., 2009). The PI infected animal should be of greatest concern within zoological collections because they may appear healthy while concurrently exposing multiple animals within the zoo to the virus. Continual viral shedding of a PI animal, combined with the possibility of minimal outward signs, makes it difficult to detect the source of infection in exotic species. This may also allow infection of hosts this virus has never before encountered. Persistent infections are often associated with decreased fertility, immunosuppression, stunted growth, and secondary infections (Potgieter, 1995; Brock, 2003). Decreased fertility is of extreme concern in zoological collections attempting to breed endangered wildlife because of the limited number of available breeding stock. Simply put, endangered species cannot afford to lose breeding specimens to viral diseases that may be preventable.

The cytopathic BVDV biotype can also be isolated from PI animals. Typically infection with cytopathic strains alone cause an acute phase disease, with rapid onset of clinical signs, patient debilitation, and death. Cytopathic strains are characterized by unrestricted viral replication, producing a large amount of virus that enters the environment, but that may be self-limiting due to resultant mortality (Peterhans et al., 2010). The cytopathic biotype develops from mutations of the non-cytopathic strain, include recombination with host cell mRNA, gene translocation and duplication, and point mutation (Brock, 2003; Peterhans et al., 2010). Cytopathic BVD viruses usually fail to establish chains of infection due to death of the infected animal, and are generally considered unable to cause persistent infection, despite isolation from PI animals (Brock, 2003). In a zoo, disease conditions like diarrhea will often lead to isolation of the ill animal from herd members not showing signs of disease as diagnostic testing is performed by veterinary staff. Assuming that isolation is prompt, this may reduce risk of exposure of other animals to cytopathic virus strains until a diagnosis is achieved.

Superinfection of PI animals with a cytopathic strain may trigger mucosal disease (MD) (Brock, 2003; Nelson et al., 2008). In a domestic cattle herd, MD is characterized by a relatively low morbidity and high case fatality between the age of 6 months and 2 years. It is unknown if exotic ruminants will develop MD with the same associated clinical signs as domestic animals. Typical gross lesions in domestic species include extensive mucosal ulceration primarily within the gastrointestinal tract, with resultant diarrhea, weight loss, and wasting (Brock, 2003; Nelson et al., 2008). The variability of clinical signs for BVDV in exotic ruminants complicates diagnosis and may result in infections being overlooked because of the lack of pathognomonic or any clinical signs.

## Control of BVDV infection

In a zoo, the clinical signs of diarrhea and weight loss associated with MD will result in animals being held off displays, quarantined, and diagnostic testing performed to determine the cause. Unfortunately, by the time clinical signs are observed, the virus has already contaminated the environment. To reduce possible transmission, all incoming animals should be quarantined appropriately for their species and tested for BVDV. The identification of asymptomatic PI individuals that are new additions, as well as those existing within the collection will help to control new sources of infection. Asymptomatic PI individuals present the greatest threat to zoo collections, especially to mixed species collections or those utilizing rotational exhibits. The focus for management of BVDV in zoos should be determining infected, especially PI, individuals and taking appropriate steps to prevent and control the spread of the virus. While vaccination is utilized to prevent fetal infections in domestic cattle, vaccination for BVDV has not been well studied and remains unproven in exotic species. In addition, the diagnostic tests used for domestic cattle likely have not been validated in exotic ruminants, and as said before, there is no pathognomonic histopathologic lesion described for BVDV (BVD, 2013). Definitive diagnosis can only be reliably made based on virus isolation or demonstration of the virus within tissues (BVD, 2013).

Serological surveys have been performed in wildlife and zoological collections in an attempt to screen for disease conditions. A recent study, evaluating the seroprevalence for BVDV in a zoo in Kuwait, had an overall prevalence of 5.3% in the bovids and cervids evaluated, with prevalence as high as 60% in Sitatunga (*Tragelaphus spekii*). In that same study Axis deer, Barbary sheep, Water deer, Dorcas and Fallow deer showed no evidence of antibodies in blood sera (Uttenthal et al., 2005). A similar 2011 study of 163 animals, composed of 39 Cameroon sheep (*Ovis ammon aries*), 11 Barbary sheep, (*Ammotragus lervia*), 57 pygmy goats (*Capra hircus*), nine Angora goats (*Capra hircus*), 21 mountain goats (*Capra aegagrus-aegagrus*), seven llamas (*Lama glama*), eight Persian goitred gazelle (*Gazella subgutturosa subgutturosa*), seven Caspian red deer (*Cervus elaphus maral*), two fallow deer (*Dama dama*), and two camels (*Camelus dromedarius*) in two Turkish zoos showed negative serum antibodies to BVDV (Yeşilbag et al., 2011). A third 2011 study of archived and fresh samples from eight different European zoos utilizing cell culture and antibody enzyme-linked immunosorbent assay (ELISA) evaluations demonstrated detection of BVDV antibodies in 23.3% (21/90) of the animals evaluated (Probst et al., 2011). Because PI individuals occur due to failure of the immune system to respond appropriately to infection by BVDV, serology has its place for survey of populations, but also has substantial shortcomings in identifying all infected individuals in a closed herd in an attempt to eradicate the virus. PI individuals may have no detectable antibody titer and thus will not be identified if this is the sole form of screening utilized in a zoo. Not only can PI individuals screened serologically remain undetected, they serve as a nidus of infection unless they are identified.

Serologic testing for domestic animals is relatively easy, however simply obtaining a blood sample for serology from most exotic ruminants generally involves the use of special restraint chutes and/or general anesthesia which present the additional risks of chute trauma or death due to anesthesia complications. Combination testing may be considered more reliable for sensitive wildlife that can only be sampled on one or limited occasions due to these risks. Use of a combination of antigen-capture ELISA (ACE) on haired skin with Real Time-PCR (RT-PCR) on whole blood (buffy coat, collected in EDTA) and antibody detection via serum neutralization has the greatest likelihood of identifying PI specimens and those that may be transiently infected (Brock, 2003; Walz et al., 2010; BVD, 2013). Haired skin should ideally be taken from the ear or caudal tail fold, however the structure of the animal being evaluated and public perception of potential permanent marks, as from an ear sample, must be considered with zoo animals (Walz et al., 2010; BVD, 2013). An animal that is positive on both ACE on haired skin, as well as RT-PCR on whole blood, but is negative on serology is considered highly suspicious of being a PI and should be isolated and undergo repeat testing 4–6 weeks later to confirm positive status (Brock, 2003; Walz et al., 2010; BVD, 2013). This follow up testing may prove to be problematic with especially sensitive zoo species, but is essential for identification of PI specimens. The risk of anesthesia must be weighed against the benefits of identifying potential disease exposure for the entire collection. Animals that are positive on RT-PCR and have positive serum titers may be considered transiently infected. These individuals need to be isolated and monitored closely for the development of clinical signs.

Pregnant females with a screening serum antibody titer to BVDV may have been exposed to the virus within the first trimester of pregnancy and as a result be carrying a PI fetus. These animals should be quarantined until the offspring is born then thoroughly screened for BVDV. The offspring should be tested for persistent infection via whole blood RT-PCR in combination with ACE or immunohistochemistry on a haired skin sample (Walz et al., 2010; BVD, 2013). Because this methodology detects actual viral antigen, not antibodies, the presence of virus can be determined in the presence of maternal antibodies if the offspring is sampled after ingestion of colostrum (Walz et al., 2010; BVD, 2013). It is also important to remember that BVDV has been isolated from commercial fetal calf plasma. If plasma is utilized in place of colostrum for neonates, or serum is used in reproduction techniques in adults, it can serve as a source of infection (Brock, 2003).

Because of risk of transmission, it is not wise to introduce any viremic animals to others that may be in the first trimester of gestation (BVD, 2013). Depending on the species, culling or complete isolation with assisted reproduction or artificial insemination techniques followed by thorough screening of offspring in the event of an endangered species, may be the most practical means of preventing the spread of the virus.

## Conclusion

BVDV is a virus capable of infecting exotic ruminants, many of which are commonly housed in zoos. Animals persistently infected with BVDV present the greatest danger to a zoological collection. Because there is no cure for BVDV, management practices in zoo collections must focus on detection of PI individuals and the prevention of the spread of the virus. Veterinarians must realize that while convenient and economical, serologic testing alone is not sufficient to rule out BVDV infection in zoo animals. Combination testing utilizing ACE from haired skin with RT-PCR has the greatest likelihood of identifying PI specimens and those that may be transiently infected. This should serve as the current “Gold Standard” for thorough survey of zoological collections.

### Conflict of interest statement

The authors declare that the research was conducted in the absence of any commercial or financial relationships that could be construed as a potential conflict of interest.
